# Instruments for measuring emotional assessment in hospital nursing care: a scoping review

**DOI:** 10.1590/0034-7167-2025-0234

**Published:** 2026-08-03

**Authors:** Frederico Marques Andrade, Lanuza Borges Oliveira, Patrícia Pereira Alves, Maria Eduarda Borges Rodrigues, Carla Silvana de Oliveira e Silva

**Affiliations:** I Universidade Estadual de Montes Claros. Montes Claros, Minas Gerais, Brazil; II Afya Centro Universitário Montes Claros. Montes Claros, Minas Gerais, Brazil

**Keywords:** Nursing Staff, Quality of Life, Burnout Professional, Surveys and Questionnaires, Scoping Review., Personal de Enfermería, Calidad de Vida, Agotamiento Profesional, Encuestas y Cuestionarios, Revisión de Alcance.

## Abstract

**Objectives:**

to analyze the instruments used by nursing professionals in hospital care to measure emotional states.

**Methods:**

this scoping review was conducted according to Preferred Reporting Items for Systematic Reviews and Meta-Analyses extension for Scoping Reviews guidelines, with a protocol registered in Open Science Framework. The search was performed in MEDLINE (via PubMed), Scopus, Web of Science, Cochrane, and Virtual Health Library. Data were organized in EndNote and analyzed using ATLAS.ti web, following Bardin’s content analysis technique.

**Results:**

of the 1,335 studies identified, 28 met the inclusion criteria. Twenty-five instruments for assessing emotional states applied to hospital nurses were mapped, most of which were not specific to nursing. Five thematic axes were identified: well-being; stress/burnout; job satisfaction; coping strategies; and interpersonal relationships.

**Conclusions:**

a wide variety of instruments were observed, with a predominance of scales developed in other contexts and a scarcity of tools validated specifically for nursing.

## RESUMEN

**Objetivos:**

analizar los instrumentos de medición del estado emocional utilizados por profesionales de enfermería en la atención hospitalaria.

**Métodos:**

revisión exploratoria realizada según la extensión de la *Preferred Reporting Items for Systematic reviews and Meta-Analyses extension for Scoping Reviews*, con un protocolo registrado en el *Open Science Framework*. La búsqueda se realizó en MEDLINE (vía PubMed), Scopus, *Web of Science*, Cochrane y la Biblioteca Virtual de Salud. Los datos se organizaron en EndNote y se analizaron mediante la plataforma web ATLAS.ti, siguiendo la técnica de análisis de contenido de Bardin.

**Resultados:**

de los 1335 estudios identificados, 28 cumplieron los criterios de inclusión. Se mapearon veinticinco instrumentos para evaluar estados emocionales aplicados a enfermeras hospitalarias, la mayoría de los cuales no eran específicos de enfermería. Se identificaron cinco ejes temáticos: bienestar; estrés/agotamiento; satisfacción laboral; estrategias de afrontamiento; y relaciones interpersonales.

**Conclusiones:**

se utilizó una amplia variedad de instrumentos, con predominio de escalas desarrolladas en otros contextos y escasez de herramientas validadas específicamente para enfermería.

## INTRODUCTION

Nursing professionals’ mental health is a critical component for healthcare quality and safety. The nature of work in hospital environments, characterized by high emotional demands, long hours, constant exposure to human distress, and situations of intense stress, makes this professional category particularly vulnerable to the development of mental disorders such as anxiety, depression, and burnout syndrome^(1,2)^. Nursing professionals experience higher rates of psychological distress compared to other professional categories, which compromises not only their individual well-being but also the quality of care provided to patients^(3)^.

Mental health’s importance is recognized globally, reflected in the United Nations Sustainable Development Goal (SDG) 3 - Good Health and Well-being, which aims to ensure healthy lives and promote well-being for all at all ages^(4)^. In this context, nursing professionals’ mental health is an indispensable condition for the sustainability of healthcare systems, ensuring more humane, safe, and effective care environments. The World Health Organization has emphasized the need for investments in healthcare workers’ mental health as an essential strategy for strengthening healthcare systems globally^(5)^.

Nursing professionals’ emotional state directly impacts the quality of care provided, patient safety, and dynamics of healthcare teams. Emotionally overburdened professionals may exhibit reduced decision-making capacity, decreased empathy, a higher risk of making care errors, and increased absenteeism and turnover rates^(6,7)^. Furthermore, mental health issues among these professionals generate significant costs for healthcare systems related to absences, treatments, and loss of productivity^(8)^.

Large-scale events, such as the COVID-19 pandemic, exacerbated the vulnerabilities of these professionals, significantly increasing the prevalence of stress, anxiety, depression, burnout, and even suicidal ideation^(9,10)^. This scenario reinforced the urgency of effective strategies for monitoring and intervening in mental health within the occupational context of nursing.

Measuring emotional states, while challenging due to its subjective nature, is fundamental for the early detection of psychological distress and the development of effective intervention strategies. The use of standardized and validated measurement instruments allows for the quantification and monitoring of professionals’ emotional well-being, providing objective data for human resource management, formulation of occupational health policies, and assessment of the effectiveness of interventions^(11,12)^. These instruments enable the identification of at-risk groups, longitudinal monitoring, and comparison between different contexts and populations.

Despite the relevance of the topic, there is an apparent dispersion in literature regarding which instruments are most used and appropriate for assessing nursing professionals’ emotional state in hospital environments. It is unclear which tools have been preferentially employed, which emotional dimensions are prioritized, and whether there are specific validated instruments for this population. A synthesis of this knowledge is essential to guide researchers, managers, and the professionals themselves in choosing the most appropriate tools, contributing to the standardization of assessments and comparability of results among studies.

## OBJECTIVES

To analyze the instruments used by nursing professionals in hospital care to measure emotional states. This initiative aligns directly with SDG 3, as promoting healthcare workers’ mental health and well-being is essential for the quality of care provided to the population.

## METHODS

### Study design

This is a scoping review, with a protocol registered in Open Science Framework (osf.io/h5vze). This type of work aims to map the main concepts, identify knowledge gaps, and clarify research areas related to the topic^(9)^; in this case, the instruments for measuring emotional responses are for nursing professionals.

This review was developed based on Preferred Reporting Items for Systematic Reviews and Meta-Analyses extension for Scoping Reviews recommendations^(10)^, using the JBI method^(11)^, based on the nine stages for conducting a scoping review, namely: 1) aligning the proposal’s objectives and questions; 2) aligning inclusion criteria with the proposal’s objectives and questions; 3) description of the screening, selection, data extraction and presentation of evidence; 4) search for evidence; 5) study/evidence selection; 6) evidence extraction; 7) evidence analysis; 8) data grouping and presentation; 9) synthesis of evidence in relation to the review’s objective through conclusions and implications of the findings.

### Search strategies

To support the objective of this study, a literature review on the subject was previously conducted in databases (Latin American and Caribbean Literature in Health Sciences, Web of Science (WoS), and MEDLINE via the National Library of Medicine (PubMed)), which did not reveal any reviews with the same characteristics, highlighting the novelty of this research.

Based on the methodological requirement of the first stage, the PCC strategy was used to identify the study question, being, for the purposes of this study: P (Population) - nursing professionals; C (Concepts) - instruments for measuring emotional issues; and C (Context) - healthcare services in tertiary care. Thus, for the product of this research, the guiding question was: “what are the characteristics of the instruments that measure emotional issues in nursing professionals in the context of healthcare services in tertiary care?”. Inclusion criteria were defined based on the PCC strategy: Population - articles involving research whose sample consisted of nursing professionals at their different professional levels (not exclusively); Concept - articles that subjected nursing professionals to the measurement of emotional conditions using validated instruments; Context - articles that measured professionals in care and management roles in hospital services, using Brazilian conceptualization and tertiary healthcare services. Quantitative studies of any design published in full in Portuguese, Spanish, and/or English until May 2024 were eligible for the database. Editorials, preprint articles, books, letters, monographs, dissertations, theses, blogs, theoretical articles, and reflection articles were excluded, given the scope of the review.

A search strategy was conducted between September 2023 and May 2024 in the MEDLINE, PubMed, Scopus, WoS, Cochrane, and Virtual Health Library databases. In the initial stage, terms from titles and abstracts related to the PCC (in the Cumulative Index to Nursing and Allied Health Literature and MEDLINE databases) were identified. For the strategy, the Boolean operators OR and AND were used, referring to each item of the PCC strategy, represented by controlled terms (Chart 1). Uncontrolled terms were added to increase the universe of the initial working database.

**Chart 1:** Search terms used and main strategy based on the Population, Concept, Context strategy, Montes Claros, Minas Gerais, Brazil, 2025

PCC	Controlled vocabularies	Uncontrolled vocabularies
Population	Nursing professionals	*Enfermeiras de Cabeceira; Enfermeiros de Cabeceira*; Nurse Practitioners; Nurses; Nursing, Team; *Enfermeras Practicantes; Enfermeros Diplomados; Personal de Enfermería Diplomado*.
Concepts	Emotional exhaustion	*Síndrome do Esgotamento*; Burnout, Psychological; Stress, Psychological*; Agotamiento Psicológico; Desgaste Psicológico; Desgaste Emocional; Síndrome de Burnout;* Burn-out; Burnout.
Context	Hospitalization	*Internação Hospitalar*; Health Services; Tertiary Healthcare; Hospital; Hospitalization; *Hospitalización; Internación Hospitalaria*.
**Featured search strategy:** (*Nurses OR* “*Nursing, Team*”) *AND* (“*Stress, Psychological*”) *AND* (“*Health Services*” *OR* “*Tertiary Healthcare*” *OR Hospital*).		

*PCC - Population, Concept, Context.*

**Figure 1:**
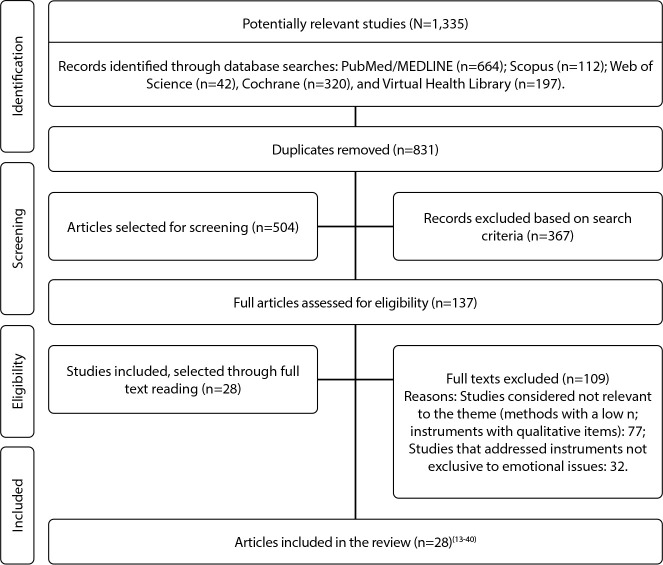
Preferred Reporting Items for Systematic Reviews and Meta-Analyses extension for Scoping Reviews flowchart showing the study selection process, Montes Claros, Minas Gerais, Brazil, 2025

To ensure that the construction of this search strategy resulted in consistent data with minimal losses, the six Peer Review Electronic Search Strategy 2015 version guidelines were used^(12)^. Following search strategy validity, all studies were analyzed by two independent reviewers. Selection inconsistencies were analyzed by a third reviewer independently to confirm eligibility. The selected studies were identified and exported to EndNote^®^, a reference manager that removed duplicates and standardized them throughout the selection process. Figure 1 shows the study selection process.

### Data extraction and quality assessment

A full-text reading of all the reserved material and all the included studies was carried out; this reading aimed to answer the review question and extract the data of interest. For this stage, a Microsoft Excel^®^ spreadsheet, developed by the authors and designed according to the following variables, was used: author; title; year of publication; country of data collection; country of publication of the study; objectives; main findings; and level of evidence. These variables were established based on JBI criteria^(11)^. It is noteworthy that these support the items present in the organization proposed by EndNote^®^. Regarding the results, the selected articles were organized, analyzed, coded, and categorized with the aid of ATLAS.ti web version 23. It is emphasized that the methodological adherence to Bardin’s content analysis^(13)^ was the route followed for the analysis of results.

## RESULTS

The search identified 1,335 potentially relevant studies in the databases, of which 831 duplicates were removed. Subsequently, 504 publications were analyzed based on their titles and abstracts, and 367 studies were excluded for not meeting the inclusion criteria. In addition, 137 articles were fully assessed for eligibility. Finally, 28 articles were selected^(14-41)^.

Most studies (Chart 2) originated from publications in the United States of America (USA) (n=10)^(15,17,20,21,24,27,29,32,40,41)^ and in England (n=7)^(25,26,34,35,37-39)^, followed by Brazil (n=3)^(22,28,31)^ and China (n=2)^(16,33)^. The publication period for these publications was from 1996 to 2021, with the majority concentrated in the last ten years^(14-18,20-27,29,31-34,37,38)^ (n=20). All studies are characterized as cross-sectional, and most fall within level V evidence^(42)^ (n=26). To assess the nursing staff’s emotional well-being, some studies^(15,19,22,26,29,30,36,39-41)^ used validated instruments specifically designed for the nursing staff (n=10).

**Chart 2:** Articles used for this study, Montes Claros, Minas Gerais, Brazil, 2025

Ref.	Author(s)	Title	Country, year	Study design, level of evidence	Instrument(s) used
13	Labrague LJ, Los Santos JAA	Fear of COVID-19, psychological distress, work satisfaction and turnover intention among frontline nurses	Philippines, 2021	Cross-sectional study, level V	Job Content Questionnaire, Fear of COVID-19 Scale, Job Satisfaction Scale
14	Ko W, Kiser-larson N	Stress Levels of Nurses in Oncology Outpatient Units	United States of America, 2016	Cross-sectional study, level V	Nursing Stress Scale
15	Li J, Su Q, Li X, Peng Y, Liu Y	COVID-19 negatively impacts on psychological and somatic status in frontline nurses	China, 2021	Cross-sectional study, level IV	Impact Event Scale, Self-Rating Anxiety Scale
16	Kelly LA, Lefton C	Effect of Meaningful Recognition on Critical Care Nurses’ Compassion Fatigue	United States of America, 2017	Cross-sectional study, level IV	Professional Quality of Life Scale
17	Okwaraji F, En A	Burnout and psychological distress among nurses in a Nigerian tertiary health institution	Nigeria, 2014	Cross-sectional study, level V	Maslach Burnout Inventory, General Health Questionnaire-12
18	Milutinović D, Golubović B, Brkić N, Prokeš B	Professional stress and health among critical care nurses in Serbia	Croatia, 2012	Cross-sectional study, level V	Nursing Stress Scale
19	Zaghloul MS, Saquib J, AlMazrou A, Saquib N	Mental Health Status of Expatriate Nurses in Northcentral Saudi Arabia	United States of America, 2019	Cross-sectional study, level V	Depression, Anxiety and Stress Scale-21
20	Kellogg MB, Knight M, Dowling JS, Crawford SL	Secondary Traumatic Stress in Pediatric Nurses	United States of America, 2018	Cross-sectional study, level V	Secondary Traumatic Stress Scale, Depression, Anxiety and Stress Scale-21
21	Portero de la Cruz S, Vaquero Abellán M	Professional burnout, stress and job satisfaction of nursing staff at a university hospital	Brazil, 2015	Cross-sectional study, level V	Nursing Stress Scale, Maslach Burnout Inventory, *Cuestionario Font Roja de Satisfacción Laboral*
22	Mushtaq M, Sultana S, Imtiaz I	The Trauma of Sexual Harassment and its Mental Health Consequences Among Nurses	Pakistan, 2015	Cross-sectional study, level V	Depression, Anxiety and Stress Scale-21
23	Yoshizawa K, Sugawara N, Yasui-Furukori N, Danjo K, Furukori H, Sato Y, Tomita T, Fujii A, Nakagam T, Sasaki M, Nakamura K	Relationship between occupational stress and depression among psychiatric nurses in Japan	United States of America, 2016	Cross-sectional study, level V	General Health Questionnaire-12, Center for Epidemiologic Studies - Depression
24	Karsavuran S, Kaya S	The relationship between burnout and mobbing among hospital managers	England, 2017	Cross-sectional study, level V	Leymann Inventory of Psychological Terrorization, Maslach Burnout Inventory
25	Vandenbroeck S, Van Gerven E, De Witte H, Vanhaecht K, Godderis L	Burnout in Belgian physicians and nurses	England, 2017	Cross-sectional study, level V	Nursing Work Index-Revised, The Ways of Coping Checklist, Maslach Burnout Inventory
26	Ceravolo D, Raines DA	The Impact of a Mindfulness Intervention for Nurse Managers	United States of America, 2019	Cross-sectional study, level V	Professional Quality of Life Scale
27	Silva AA, Souza JM, Fischer FM	Health-related quality of life and working conditions among nursing providers	Brazil, 2010	Cross-sectional study, level V	Job Satisfaction Scale, Effort-Reward Imbalance, Short-Form Health Survey-36
28	Qin Z, Zhong X, Ma J, Lin H	Stressors affecting nurses in China	United States of America, 2016	Cross-sectional study, level V	Nursing Stress Scale
29	Kawaguchi Y, Toyomasu K, Yoshida N, Baba K, Uemoto M, Minota S	Measuring job stress among hospital nurses: an attempt to identify biological markers	Japan, 2007	Cross-sectional study, level V	Job Satisfaction Scale, Nursing Stress Scale
30	Ramos FRS, Brehmer LCF, Dalmolin GL, Silveira LR, Schneider DG, Vargas MAO	Association between moral distress and supporting elements of moral deliberation in nurses	Brazil, 2020	Cross-sectional study, level V	Moral Distress Scale
31	Jang MH, Gu SY, Jeong YM	Role of Coping Styles in the Relationship Between Nurses’ Work Stress and Well-Being Across Career	United States of America, 2019	Cross-sectional study, level V	Nursing Stress Scale, The Ways of Coping Checklist Scale, Ryff’s Psychological Wellbeing Scale
32	Liu S, Zhao N, Xie Y, Wang S, Ouyang X, Guo F, Gong Z, Zhou Q, Li J	Psychological status of the staff in a general hospital during the outbreak of coronavirus disease 2019 and its influential factors	China, 2020	Cross-sectional study, level V	Depression, Anxiety and Stress Scale-21, The Ways of Coping Checklist Scale, Professional Quality of Life Scale
33	Asgari S, Shafipour V, Taraghi Z, Yazdani-Charati J	Relationship between moral distress and ethical climate with job satisfaction in nurses	England, 2019	Cross-sectional study, level V	Moral Distress Scale, Job Satisfaction Scale
34	Fagin L, Carson J, Leary J, De Villiers N, Bartlett H, O’Malley P, West M, McElfatrick S, Brown D	Stress, coping and burnout in mental health nurses: findings from three research studies	England, 1996	Cross-sectional study, level V	General Health Questionnaire-12, Maslach Burnout Inventory, De Villiers, Carson & Leary Stress Scale, The Ways of Coping Checklist Scale
35	Pinikahana J, Happell B	Stress, burnout and job satisfaction in rural psychiatric nurses: a Victorian study	Australia, 2004	Cross-sectional study, level V	Maslach Burnout Inventory, Nursing Stress Scale, Job Satisfaction Scale
36	Wang J, Okoli CTC, He H, Feng F, Li J, Zhuang L, Lin M	Factors associated with compassion satisfaction, burnout, and secondary traumatic stress among Chinese nurses in tertiary hospitals: A cross-sectional study	England, 2020	Cross-sectional study, level V	Professional Quality of Life Scale
37	Ohnishi K, Kitaoka K, Nakahara J, Välimäki M, Kontio R, Anttila M	Impact of moral sensitivity on moral distress among psychiatric nurses	England, 2019	Cross-sectional study, level V	Moral Sensitivity Questionnaire, Moral Distress Scale
38	Chang EM, Bidewell JW, Huntington AD, Daly J, Johnson A, Wilson H, Lambert VA, Lambert CE	A survey of role stress, coping and health in Australian and New Zealand hospital nurses	England, 2007	Cross-sectional study, level V	Nursing Stress Scale, The Ways of Coping Checklist Scale, Short-Form Health Survey-36
39	Sawatzky JA	Stress in critical care nurses: actual and perceived	United States of America, 1996	Cross-sectional study, level V	Critical Care Nursing Stress Scale, Perceived Stress Scale
40	Fukuda H, Ichinose T, Kusama T, Yoshidome A, Anndow K, Akiyoshi N, Shibamoto T	The relationship between job stress and urinary cytokines in healthy nurses: a cross-sectional study	United States of America, 2008	Cross-sectional study, level V	Nursing Stress Scale, Profile of Mood States

Twenty-five instruments for assessing emotional conditions were used in the studies (Chart 3), most of which were developed and validated in the 1990s (n=12). The most frequently found instruments in studies with nursing professionals working in hospital environments were: the Nursing Stress Scale (NSS) (n=9); the Maslach Burnout Inventory (MBI) (n=6); the Job Satisfaction Scale (JSS) and The Ways of Coping Checklist (WCCL) (both with n=5); the Depression, Anxiety and Stress Scale-21 (DASS-21) and the Professional Quality of Life Scale (ProQol) (both with n=4); the Moral Distress Scale (MDS) and the General Health Questionnaire-12 (GHQ-12) (both with n=3); and the Short-Form Health Survey-36 (SF-36) (n=2). A portion of the studies^(15,17,19,20,23,27,29,31,37)^ applied a single instrument to achieve its objectives (n=9). The instruments used individually were the ProQol, MDS, NSS, and DASS-21. Most of the instruments’ structure are multidimensional in nature (n=20). All instruments use the Likert-type scale as the response method.

**Chart 3:** Instruments used in the studies of this research, Montes Claros, Minas Gerais, Brazil, 2025

	Instrument	How many times it was used	Year of elaboration	Country of origin	Number of structural dimensions	Number of items to be answered	Assessment areas
1	Nursing Stress Scale	9	1974	Canada	2	34	Stress: workload, death and dying, preparation, support from colleagues, uncertain treatment, medical conflict, nursing conflict. Work organization.
2	Maslach Burnout Inventory	6	1981	United States of America	3	22	Emotional exhaustion, depersonalization, personal accomplishment at work.
3	Job Satisfaction Scale	5	1985	United States of America	9	36	Compensation, promotion, supervision, benefits, rewards, operating conditions, colleagues, nature of work, and communication.
4	The Ways of Coping Checklist	5	1980	United States of America	2	66	Problem-focused coping: planning: Developing an action plan to directly address the problem. Active problem-solving: taking concrete stages to resolve or mitigate the stressful situation. Seeking instrumental support: seeking practical help or guidance to deal with the problem/emotion-focused coping. Seeking emotional support: seeking empathy, understanding, or emotional support from others. Acceptance: accepting the reality of the stressful situation without necessarily trying to change it. Activity suppression: reducing activities or distractions to cope with the emotional load.
5	Depression, Anxiety and Stress Scale-21	4	1995	Australia	3	21	Depression (feelings of sadness, hopelessness, lack of interest or pleasure in daily activities, among other symptoms associated with depression), anxiety (experiences of nervousness, restlessness, excessive worry, fear or apprehension about the future) and stress (inability to relax, irritability, impatience, feelings of being overwhelmed).
6	Professional Quality of Life Scale	4	1995	United States of America	3	28	Compassion satisfaction (items related to job satisfaction, sense of purpose, and personal fulfillment), compassion fatigue (emotional and psychological exhaustion resulting from continuous involvement in situations of distress and trauma. Includes items such as symptoms of stress, burnout, and emotional exhaustion), and burnout.
7	Moral Distress Scale	3	1984	United States of America	3	38	Causes, manifestations, moral consequences. Ethical conflict, impotence and frustration, moral discomfort, recurring ethical dilemmas, professional autonomy.
8	General Health Questionnaire-12	3	1979	United Kingdom	3	12	Social dysfunction, anxiety and depression, loss of confidence.
9	Short-Form Health Survey-36	2	1992	United States of America	8	36	Physical functioning, role-physical, bodily pain, general health status, vitality, social functioning, role-emotional, mental health
10	Critical Care Nursing Stress Scale	1	1996	Canada	4	40	Interpersonal relationships, patient care, knowledge and skills, physical environment.
11	Moral Sensitivity Questionnaire	1	1993	Sweden	6	30	Interpersonal orientation, structuring moral meaning, expressing benevolence, modifying autonomy, experiencing moral conflict, confidence in professional knowledge.
12	Job Content Questionnaire	1	1979	United States of America	3	49	Psychological demands, decision latitude, social support.
13	Nursing Work Index-Revised	1	2001	United States of America	6	60	Autonomy, control over work environment, management support, nurse-physician relations, job satisfaction, intention to leave their job.
14	Leymann Inventory of Psychological Terrorization	1	1990	Sweden	1	45	The instrument assesses exposure to psychological terror behaviors in the workplace. Assessment areas include various psychological terror behaviors such as social isolation, defamation, discrimination, and other types of psychological harassment.
15	Secondary Traumatic Stress Scale	1	1995	United States of America	3	17	Intrusive symptoms, avoidance symptoms, arousal symptoms.
16	Impact Event Scale	1	1997	United States of America	3	22	Intrusion (refers to the presence of unwanted thoughts and images related to the traumatic event), avoidance (assesses avoidance behaviors, such as avoiding thoughts and feelings related to the event), hyperarousal (measures symptoms of hyperarousal, such as irritability, difficulty concentrating, and hypervigilance).
17	De Villiers, Carson & Leary Stress Scale	1	1996	United States of America	3	30	Emotional exhaustion, depersonalization, personal fulfillment.
18	Effort-Reward Imbalance	1	1996	Germany	3	16	Effort: assesses the perceived level of psychological and physical demands at work. Rewards: measures perceived rewards, including salary, promotions, recognition, and job security. Imbalance: reflects the comparison between effort invested and rewards received.
19	Self-Rating Anxiety Scale	1	1971	United States of America	1	20	Severity of anxiety symptoms.
20	Center for Epidemiologic Studies - Depression	1	1977	United States of America	1	20	Assesses a variety of symptoms related to depression, including feelings of sadness, lack of interest, loss of appetite, and sleep difficulties.
21	Fear of COVID-19 Scale	1	2020	Turkey	1	7	Health concerns: assesses fear related to one’s own health and the possibility of contracting COVID-19. Anxiety about contagion: explores concern and anxiety regarding the possibility of transmitting or contracting the virus, either to oneself or to others. General psychological impact: includes items that address the broader emotional impact of the pandemic, such as anxiety, constant worry, and psychological stress. Fear of dying due to COVID-19: focuses specifically on the fear of death associated with infection by the virus.
22	Perceived Stress Scale	1	1983	United States of America	1	14	Sense of unpredictability: assesses the degree to which an individual’s life is perceived as unpredictable or uncontrollable. Feeling of control: addresses the individual’s perception of the control they believe they have over life situations. Perceived overload: refers to the perception of being overwhelmed by demands, responsibilities, or pressures in daily life.
23	Profile of Mood States	1	1971	United States of America	6	65	Tension-anxiety: assesses the degree of nervousness, tension, and anxiety the person is experiencing. Depression-dejection: measures feelings of sadness, discouragement, and hopelessness. Anger-hostility: assesses a person’s propensity to become irritated, angry, or frustrated. Vigor-activity: refers to physical and mental energy and disposition. Fatigue-inertia: assesses the level of perceived fatigue and tiredness. Confusion-bewilderment: measures the degree of confusion, disorientation, and lack of mental clarity.
24	*Cuestionario Font Roja de Satisfacción Laboral*	1	1988	Spain	9	24	Job satisfaction, work stress, professional competence, work pressure, career advancement, interpersonal relationships with superiors, interpersonal relationships with colleagues, extrinsic characteristics of social status, and monotony at work.
25	Ryff’s Psychological Wellbeing Scale	1	1980	United States of America	6	84	Autonomy: assesses the sense of independence and self-determination. Positive relations with others: positive engagement with others and healthy interpersonal relationships. Environmental mastery: a sense of mastery and competence in managing the environment. Personal growth: continuous pursuit of personal growth and development. Purpose in life: having meaningful goals and a clear sense of direction in life. Self-acceptance: acceptance and respect for oneself, including both positive and negative aspects.

Five groupings of similarity emerged from the analysis of the content of the dimensions and assessment items of the instruments, coded as: professional well-being; stress and burnout; job satisfaction and dissatisfaction; coping strategies; working conditions and interpersonal relationships.

## DISCUSSION

The reviewed studies point to several important factors for understanding nursing professionals’ emotional state. Some discussions of these findings will be systematized to understand this topic, which is largely based on the use of instruments for its measurement, since using validated instruments to understand subjective circumstances, such as emotional ones, becomes essential for its comprehension and analysis.

The USA stands out as a place for publishing articles in this field. This characteristic is related to factors stemming from its scientific, social, and economic culture. Historically, the USA has led in promoting health research through financial incentives and a large number of scientific dissemination channels^(43)^. This encouragement may be related to aspects ranging from the contemporary construction of American nationalism, based on policies such as America First^(44)^, to economic concerns about American healthcare system costs. Nursing is the largest category in a healthcare system. Thus, constant emotional monitoring means fewer absences and losses in the provision of healthcare services^(45)^.

The results of this review indicate that the ten years prior to this research (2014-2023) were very representative in the application of emotional assessment instruments among nursing professionals in a hospital environment. Several events contribute to the increase in research in the area of mental health among nursing professionals. We should begin by mentioning the increase, during this period, in the complexity of care to which these professionals are subjected in the provision of their services, which increases workload, feelings of anxiety, and stress^(46)^. Furthermore, all these situations were exacerbated by the emergence of the COVID-19 pandemic at the end of 2019, which caused serious emotional distress for nursing professionals worldwide^(47)^. This event is so important to this topic that it culminated in the development of a specific instrument, Fear of COVID-19 Scale, to assess the emotional state of healthcare professionals treating patients with this diagnosis^(14)^.

The studies mostly used instruments developed in the 1990s. This decade is marked in Brazil and worldwide by theoretical, diagnostic, and ideological evolutions, which directly contributed to the emergence of concerns about mental health and the consequent emergence of instruments. The demystification and reduction of stigma for patients with mental disorders, advances in research and treatment in the area, activism and advocacy for patients (in Brazil, the enormous discussion of the Psychiatric Reform stands out), and the increase in diagnostic possibilities were fundamental pillars for this occurrence^(48)^. All of these movements have impacted not only mental healthcare, but also assessments of emotional well-being among healthcare professionals.

This review resulted in a series of instruments used to assess emotional conditions in nursing professionals in hospital environments. The study highlighted the NSS, specific to the nursing field, as well as the MBI (n=6), the JSS, the DASS-21, and the ProQol (both with n=4). It also emphasized instruments specific to the nursing team. Instruments validated exclusively for specific categories have become fundamental for understanding the phenomenon in specific populations^(49)^. Only specialized instruments can measure unique realities not evident in general contexts.

Analyzing the dimensions and nature of the instruments was essential to understanding the contexts and universes considered when assessing nursing professionals’ emotional conditions. Clusters of coded similarity emerged, which we will detail from this point forward for a more thorough understanding.

Tools more focused on professional well-being are considered multidimensional, meaning they take into account that emotional issues are consequences of various factors, both internal and external to the person. In contemporary times, evidence for addressing emotional issues highlights the importance of integrated and multidimensional perspectives and actions^(50)^. Several studies^(15,17,19,20,23,27,28,32,37,39)^ have used this type of instrument, with dimensions that address different areas of focus.

The instruments revealed for this category are the ProQol, the SF-36, the Ryff’s Psychological Wellbeing Scale, the NSS, and the DASS-21. It should be added that the multidimensional approach of this instrument category allowed studies^(15,17,19,20,23,27,29,37)^ that used a single instrument. These studies considered that its isolated application reveals sufficient results for analyzing the nature of emotional issues.

“Stress and burnout” was another grouping. The instruments that focused their results on analyzing the most anticipated consequences of emotionally debilitating situations, stress, and burnout stand out within this thematic category. Professionals working in hospital units, environments with high psychological demands and low control over these demands, are constantly experiencing burnout and occupational stress^(46)^.

The instruments that serve this purpose are the MBI, the MDS, the GHQ-12, and the WCCL. These instruments have been used in studies^(18,22,25,26,31,34,35,36,38)^ that, among other objectives, sought to detect evidence of established emotional issues (in this case, stress and burnout). Depreciative emotional situations, such as stress and burnout, in nursing professionals, have catastrophic impacts in various dimensions, whether in professionals’ personal lives, their job security, or patient safety^(13)^.

“Job satisfaction and dissatisfaction” was another grouping of instruments that emerged from certain studies analyzed^(14,16,22,26,28,36)^. This encompasses instruments that can assess aspects of job satisfaction among hospital nursing professionals while performing their duties. In other words, these instruments can assess phenomena that occur while providing nursing services to hospitalized patients and that generate emotional changes. The determinants of job satisfaction or dissatisfaction are diverse, multifactorial, and dynamic, influenced by their environment, working conditions, and the characteristics that workers possess to cope with the adversities of their environments. Dissatisfaction is related to the experience of maladjustment between mental and physical components, distress, anxiety, and difficulties in work organization, not always explicitly stated by workers. Satisfaction, on the other hand, is related to the meaning and value attributed to work by individuals, the ability to adapt to the content of productive activities, interpersonal relationships, the quality of a task performed, and well-being^(50)^.

The instruments that fall into this category were the JSS, the Nursing Work Index-Revised (NWI-R), the Job Content Questionnaire (JCQ), and the *Cuestionario Font Roja de Satisfacción Laboral*.

“Coping strategies” was another emerging category from the content analysis of the instrument dimensions in the studies^(26,32,39)^. This category is important because it points to the emotional changes resulting from mechanisms to cope with, control, or minimize the consequences of anxiety and stress situations already present in nursing workers in hospital environments. The instrument that fit into this category was the WCCL. Note that this instrument also emerged in the stress and burnout category, since it is an instrument that points to emotional effects and verifies coping methods. As a coping characteristic verified by the instrument, we can highlight resilience and coping.

Resilience results from the interaction between individual and environmental attributes (family, social, cultural) to provide a capacity for response and overcoming challenges. Resilience encompasses forms of resistance to stress, while coping represents the ability to face stress and promote positive adaptation. Coping occurs at a specific moment, and resilience over time. Nursing professionals with higher levels of self-efficacy and resilience are less likely to develop mental disorders such as depression and burnout^(39)^.

Another category in this study for understanding the instruments was “working conditions and interpersonal relationships”. This takes into account, among other factors, external situations of professionals in work environments, such as the physical structure, social support, and interpersonal relationships between nursing staff and other categories that make up the hospital service delivery environment. Several external situations negatively impact nursing professionals’ emotional well-being. Work overload, lack of adequate structural and technological resources, and communication difficulties are identified as points to be investigated.

Actions should focus on these contexts, and effective communication among workers can make a difference in work environments and mental health, as it enhances teamwork, participation in operational workflow decisions, and support among colleagues^(47)^. Some studies^(22,26,40,41)^ used instruments from this category, such as the Critical Care NSS, the NWI-R and the JCQ, with the latter two also being present in the job satisfaction and dissatisfaction category.

The findings of this review show that, although there is a diversity of instruments for measuring emotional states in hospital nurses, most were not developed specifically for this professional category, representing the main gap identified in this study.

The most frequently used instruments, such as the NSS, the MBI, the DASS-21, and the ProQol, encompass multiple emotional dimensions and can be applied individually or in combination, depending on the assessment objectives. This synthesis helps guide researchers and managers in choosing appropriate tools for assessing nursing professionals’ mental health, while also highlighting the urgent need for the development and validity of specific instruments that identify the particularities of nursing work in hospital environments.

Availability of specific and culturally adapted tools is essential to support institutional prevention and intervention policies, promote healthy work environments and, consequently, improve the quality and safety of care provided to patients.

### Study limitations

The limitations of this review include the restriction to articles published in Portuguese, English, and Spanish, which may have resulted in the exclusion of relevant studies in other languages. Additionally, the exclusion of grey literature, while justified by the focus on instruments published in peer-reviewed journals, may have limited the identification of tools in the validity phase. Finally, the content analysis process, although conducted rigorously, has an inherent interpretative component, the bias of which was minimized by independent analysis by multiple reviewers.

### Contributions to nursing, health, or public policy

This study makes a significant contribution to nursing, health, and public policy by systematizing and highlighting the most widely used validated instruments for assessing nursing professionals’ emotional well-being in hospital environments. By mapping thematic categories such as stress, burnout, job satisfaction, coping strategies, and working conditions, the research allows for the identification of critical dimensions that directly impact professional well-being and, consequently, the quality of care provided to patients. These findings not only strengthen the theoretical and methodological foundation of nursing as a science concerned with professionals’ mental health, but also provide technical support for the formulation of more effective institutional policies. Public health policies aimed at preventing mental illness, strengthening resilience, and promoting healthy work environments can be supported by the instruments and evidence discussed here, resulting in strategic measures for work management and valuing the nursing workforce, the largest professional category in health. Thus, this study reaffirms the relevance of systematic emotional assessment as a guiding principle for clinical, administrative, and policy decisions.

## CONCLUSIONS

This scoping review identified 25 instruments for measuring emotional states applied to hospital nursing professionals, mapped from 28 studies. The most frequently used instruments were the NSS, the MBI, the DASS-21, and the ProQOL. The analysis revealed five main thematic categories: professional well-being; stress and burnout; job satisfaction; coping strategies; and working conditions/interpersonal relationships.

The main finding is the predominance of generic instruments not specifically developed for the particularities of nursing practice, which represents a significant gap in literature. Most tools are multidimensional and date from the 1990s, indicating a need for updating and developing new instruments that consider contemporary work contexts.

The results reinforce the need for future research focused on the development and validity of specific instruments for nursing that can more accurately capture nursing’s emotional challenges. Appropriate tools are essential to support the creation of effective interventions, promote healthy work environments and, consequently, improve quality of care and patient safety, in alignment with SDG 3.

## Data Availability

The research data are available within the article.
