# Supplementary material for: Instruments for measuring emotional assessment in hospital nursing care: a scoping review
Source: Rev Bras Enferm. 2026 Aug 3;79:e20250234. doi: 10.1590/0034-7167-2025-0234 (PMC13432809; doi:10.1590/0034-7167-2025-0234)
Supplement: Translation

# Translation: Instrumentos de mensuração emocional na assistência de enfermagem hospitalar: revisão de escopo

**DOI:** 10.1590/0034-7167-2025-0234pt

**Authors:** Frederico Marques Andrade, Lanuza Borges Oliveira, Patrícia Pereira Alves, Maria Eduarda Borges Rodrigues, Carla Silvana de Oliveira e Silva

**Affiliations:** I Universidade Estadual de Montes Claros. Montes Claros, Minas Gerais, Brasil; II Afya Centro Universitário Montes Claros. Montes Claros, Minas Gerais, Brasil

**Keywords:** Profissionais de Enfermagem, Qualidade de Vida, Esgotamento Profissional, Inquéritos e Questionários, Revisão de Escopo.

## Abstract

**Objetivos:**

analisar os instrumentos de mensuração de estados emocionais utilizados por profissionais de enfermagem na assistência hospitalar.

**Métodos:**

revisão de escopo conduzida conforme diretrizes do *Preferred Reporting Items for Systematic reviews and Meta-Analyses extension for Scoping Reviews*, com protocolo registrado na *Open Science Framework*. A busca foi realizada na MEDLINE (via PubMed), Scopus, *Web of Science*, Cochrane e Biblioteca Virtual em Saúde. Os dados foram organizados no *EndNote* e analisados com apoio do ATLAS.ti *web*, seguindo técnica de análise de conteúdo de Bardin.

**Resultados:**

dos 1.335 estudos identificados, 28 atenderam aos critérios de inclusão. Foram mapeados 25 instrumentos de avaliação de estados emocionais aplicados em enfermeiros hospitalares, sendo a maioria não específica para enfermagem. Identificaram-se cinco eixos temáticos: bem-estar; estresse/*burnout*; satisfação laboral; estratégias de enfrentamento; relações interpessoais.

**Conclusões:**

observou-se ampla variedade de instrumentos utilizados, com predominância de escalas desenvolvidas em outros contextos e escassez de ferramentas validadas especificamente para enfermagem.

## INTRODUÇÃO

A saúde mental dos profissionais de enfermagem é um componente crítico para a qualidade e segurança da assistência à saúde. A natureza do trabalho em ambientes hospitalares, caracterizada por alta demanda emocional, longas jornadas, exposição constante ao sofrimento humano e situações de estresse intenso, torna esta categoria profissional particularmente vulnerável ao desenvolvimento de transtornos mentais, como ansiedade, depressão e a síndrome de *burnout* ^(1,2)^. Profissionais de enfermagem apresentam taxas elevadas de sofrimento psíquico quando comparados a outras categorias profissionais, o que compromete não apenas seu bem-estar individual, mas também a qualidade do cuidado prestado aos pacientes^(3)^.

O reconhecimento da importância da saúde mental é global, refletido no Objetivo de Desenvolvimento Sustentável (ODS) 3 - Saúde e Bem-Estar da Organização das Nações Unidas, que visa assegurar uma vida saudável e promover o bem-estar para todos, em todas as idades^(4)^. Nesse contexto, a saúde mental dos profissionais de enfermagem é condição indispensável para a sustentabilidade dos sistemas de saúde, garantindo ambientes de cuidado mais humanos, seguros e eficazes. A Organização Mundial da Saúde tem enfatizado a necessidade de investimentos em saúde mental dos trabalhadores da saúde como estratégia essencial para o fortalecimento dos sistemas de saúde globalmente^(5)^.

O estado emocional dos profissionais de enfermagem impacta diretamente a qualidade da assistência prestada, a segurança do paciente e a dinâmica das equipes de saúde. Profissionais emocionalmente sobrecarregados podem apresentar menor capacidade de tomada de decisão, empatia reduzida, maior risco de cometer erros assistenciais, e aumento nas taxas de absenteísmo e de rotatividade^(6,7)^. Além disso, o adoecimento mental desses profissionais gera custos significativos para os sistemas de saúde relacionados a afastamentos, tratamentos e perda de produtividade^(8)^.

Eventos de grande magnitude, como a pandemia de COVID-19, exacerbaram as vulnerabilidades desses profissionais, aumentando significativamente a prevalência de estresse, ansiedade, depressão, esgotamento e até mesmo ideação suicida^(9,10)^. Esse cenário reforçou a urgência de estratégias efetivas de monitoramento e intervenção em saúde mental no contexto ocupacional da enfermagem.

A mensuração de estados emocionais, embora desafiadora por sua natureza subjetiva, é fundamental para a detecção precoce de sofrimento psíquico e para o desenvolvimento de estratégias de intervenção eficazes. A utilização de instrumentos de medida padronizados e validados permite quantificar e monitorar o bem-estar emocional dos profissionais, fornecendo dados objetivos para a gestão de pessoas, formulação de políticas de saúde ocupacional e avaliação da efetividade de intervenções^(11,12)^. Tais instrumentos possibilitam a identificação de grupos de risco, o acompanhamento longitudinal, e a comparação entre diferentes contextos e populações.

Apesar da relevância do tema, há uma aparente dispersão na literatura sobre quais instrumentos são mais utilizados e adequados para avaliar os estados emocionais de profissionais de enfermagem no contexto hospitalar. Não está claro quais ferramentas têm sido preferencialmente empregadas, quais dimensões emocionais são priorizadas e se existem instrumentos específicos validados para esta população. Uma síntese desse conhecimento é essencial para orientar pesquisadores, gestores e os próprios profissionais na escolha das ferramentas mais apropriadas, contribuindo para a padronização de avaliações e comparabilidade de resultados entre estudos.

## OBJETIVOS

Analisar os instrumentos de mensuração de estados emocionais utilizados por profissionais de enfermagem na assistência hospitalar. Tal iniciativa se alinha diretamente à meta do ODS 3, na medida em que a promoção da saúde mental e do bem-estar dos trabalhadores da saúde é condição indispensável para a qualidade da assistência prestada à população.

## MÉTODOS

### Desenho do estudo

Trata-se de revisão de escopo, com protocolo registrado no *Open Science Framework* (osf.io/h5vze). Esse tipo de trabalho tem a finalidade de mapear os principais conceitos, identificar lacunas do conhecimento e clarear as áreas de pesquisa do tema^(9)^; neste caso, os instrumentos de mensuração emocional para profissionais de enfermagem.

Esta revisão foi desenvolvida fundamentada nas recomendações do *Preferred Reporting Items for Systematic Reviews and Meta-Analyses extension for Scoping Reviews* ^(10)^, com utilização do método JBI^(11)^, pautado nas nove etapas para a realização de uma revisão de escopo, sendo: 1) alinhamento dos objetivos e questões da proposta; 2) alinhamento dos critérios de inclusão diante dos objetivos e questões da proposta; 3) descrição da triagem, seleção, extração de dados e apresentação de evidências; 4) procura das evidências; 5) seleção dos estudos/evidências; 6) extração das evidências; 7) análise das evidências; 8) agrupamento e apresentação dos dados; 9) síntese das evidências diante do objetivo da revisão por meio de conclusões e implicações dos achados.

### Estratégias de busca

Para fundamentar o objetivo desta, previamente, realizou-se revisão de literatura acerca do assunto em bases de dados (Literatura Latino-Americana e do Caribe em Ciências da Saúde, *Web of Science* (WoS) e MEDLINE via *National Library of Medicine* (PubMed)) que não revelou revisões com as mesmas características, evidenciando o ineditismo da presente pesquisa.

Partindo-se do requisito metodológico da primeira etapa, para a identificação da questão do estudo, utilizou-se a estratégia PCC, sendo, para fins deste estudo: P (População) - profissionais de enfermagem; C (Conceitos) - instrumentos de mensuração emocional; e C (Contexto) - serviços de saúde na atenção terciária. Assim, para o produto desta pesquisa, a questão norteadora foi: “quais as características dos instrumentos que mensuram questões emocionais em profissionais de enfermagem no contexto de serviços de saúde da atenção terciária?”. Os critérios de inclusão obedecidos foram definidos a partir da estratégia PCC: População - artigos envolvendo pesquisas cuja amostra fosse composta por profissionais de enfermagem em seus diferentes níveis profissionais (não exclusivamente); Conceito - artigos que submetessem os profissionais de enfermagem à mensuração de condições emocionais por meio de instrumentos validados; Contexto - artigos que mensurassem profissionais em condições de assistência e gerência em serviços de hospitalares, utilizando conceituação brasileira e serviços terciários de assistência à saúde. Estudos quantitativos de qualquer desenho publicados integralmente nas línguas portuguesa, espanhola e/ou inglesa até maio de 2024 foram elegíveis para o banco. Houve exclusão de editoriais, artigos *preprints*, livros, cartas, monografias, dissertações, teses, blogs, artigos teóricos e artigos de reflexão, visto o objetivo da revisão de escopo.

A estratégia de busca foi realizada entre setembro de 2023 e maio de 2024 nas bases de dados MEDLINE, PubMed, *Scopus*, WoS, Cochrane, e Biblioteca Virtual em Saúde. Na etapa inicial, foi realizada a identificação de termos de títulos e resumos que se relacionam com a PCC (nas bases *Cumulative Index to Nursing and Allied Health Literature* e MEDLINE). Para a estratégia, utilizaram-se os operadores booleanos *OR* e *AND*, referentes a cada item da estratégia PCC, representados por termos controlados (Quadro 1). Os termos não controlados foram acrescidos para aumento do universo do banco inicial de trabalho.

**Quadro 1:** Termos de busca utilizados e principal estratégia com base na estratégia População, Conceito, Contexto, Montes Claros, Minas Gerais, Brasil, 2025

PCC	Vocabulários controlados	Vocabulários não controlados
População	Profissionais de enfermagem	Enfermeiras de Cabeceira; Enfermeiros de Cabeceira; *Nurse Practitioners; Nurses; Nursing, Team; Enfermeras Practicantes; Enfermeros Diplomados; Personal de Enfermería Diplomado*.
Conceitos	Esgotamento emocional	Síndrome do Esgotamento; *Burnout, Psychological; Stress, Psychological; Agotamiento Psicológico*; Desgaste Psicológico; Desgaste Emocional; Síndrome de *Burnout; Burn-out; Burnout*.
Contexto	Hospitalização	Internação Hospitalar; *Health Services; Tertiary Healthcare; Hospital; Hospitalization; Hospitalización; Internación Hospitalaria*.
**Estratégia de busca em destaque:** (*Nurses OR* “*Nursing, Team*”) *AND* (“*Stress, Psychological*”) *AND* (“*Health Services*” *OR* “*Tertiary Healthcare*” *OR Hospital*).		

*PCC - População, Conceito, Contexto.*

Para que a construção desta estratégia de busca resultasse em dados consistentes e com mínimo de perdas, utilizaram-se as seis diretrizes *Peer Review Electronic Search Strategy*, versão 2015^(12)^. Posterior à validação da estratégia de busca, todos os estudos foram analisados por dois revisores independentes. Inconsistências de seleção foram analisadas por um terceiro revisor, de forma independente, para confirmar a elegibilidade. Os estudos selecionados foram identificados e exportados para o *software* EndNote^®^, um gerenciador de referências que removeu duplicidades e padronizou as mesmas para todo o processo de seleção. A Figura 1 mostra o processo de seleção dos estudos.

**Figura 1:** Diagrama de fluxo *Preferred Reporting Items for Systematic Reviews and Meta-Analyses extension for Scoping Reviews* mostrando o processo de seleção dos estudos, Montes Claros, Minas Gerais, Brasil, 2025

### Extração de dados e avaliação de qualidade

Realizou-se a leitura na íntegra de todo o material reservado e de todos os estudos incluídos; essa leitura objetivou atender à pergunta da revisão e extrair os dados de interesse. Para esta etapa, utilizou-se planilha do *software Microsoft Excel^®^*, elaborada pelos autores e desenhada conforme as seguintes variáveis: autor; título; ano de publicação; país de coleta de dados; país de publicação do estudo; objetivos; principais achados; e nível de evidência. Essas variáveis foram estabelecidas com base nos critérios do JBI^(11)^. Ressalta-se que essas corroboram os itens presentes na organização proposta pelo *software* EndNote^®^. A respeito dos resultados, os artigos eleitos foram organizados, analisados, codificados e categorizados com o auxílio do *software* ATLAS.ti *web*, versão 23. Ressalta-se que a obediência metodológica de análise de conteúdo de Bardin^(13)^ foi a rota obedecida para a análise dos resultados.

## RESULTADOS

A busca identificou 1.335 estudos potencialmente relevantes nas bases de dados, dos quais 831 duplicatas foram removidas. Em seguida, 504 publicações foram analisadas por seus títulos e resumos, e 367 estudos foram excluídos por não atenderem aos critérios de inclusão. Além disso, 137 artigos foram totalmente avaliados para elegibilidade. Ao final, foram selecionados 28 artigos^(14-41)^.

A maioria dos estudos (Quadro 2) teve origem de publicação nos Estados Unidos da América (EUA) (n=10)^(15,17,20,21,24,27,29,32,40,41)^ e na Inglaterra (n=7)^(25,26,34,35,37-39)^, seguidos de Brasil (n=3)^(22,28,31)^ e China (n=2)^(16,33)^. O período temporal de publicação dos mesmos foi do ano de 1996 a 2021, sendo que os últimos dez anos concentram a maioria^(14-18,20-27,29,31-34,37,38)^ (n=20). Todos os estudos se caracterizam como transversais, e sua maioria se enquadra com nível de evidência V^(42)^ (n=26). Para avaliar as condições emocionais da equipe de enfermagem, alguns estudos^(15,19,22,26,29,30,36,39-41)^ utilizam instrumentos validados de forma específica para a equipe de enfermagem (n=10).

**Quadro 2:** Artigos utilizados para este estudo, Montes Claros, Minas Gerais, Brasil, 2025

Ref.	Autor(es)	Título	País, ano	Tipo de estudo, nível de evidência	Instrumento(s) utilizado(s)
13	Labrague LJ, Los Santos JAA	*Fear of COVID-19, psychological distress, work satisfaction and turnover intention among frontline nurses*	Filipinas, 2021	Estudo transversal, nível V	*Job Content Questionnaire, Fear of COVID-19 Scale, Job Satisfaction Scale*
14	Ko W, Kiser-larson N	*Stress Levels of Nurses in Oncology Outpatient Units*	Estados Unidos da América, 2016	Estudo transversal, nível V	*Nursing Stress Scale*
15	Li J, Su Q, Li X, Peng Y, Liu Y	*COVID-19 negatively impacts on psychological and somatic status in frontline nurses*	China, 2021	Estudo transversal, nível IV	*Impact Event Scale, Self-Rating Anxiety Scale*
16	Kelly LA, Lefton C	*Effect of Meaningful Recognition on Critical Care Nurses’ Compassion Fatigue*	Estados Unidos da América, 2017	Estudo transversal, nível IV	*Professional Quality of Life Scale*
17	Okwaraji F, En A	*Burnout and psychological distress among nurses in a Nigerian tertiary health institution*	Nigéria, 2014	Estudo transversal, nível V	*Maslach Burnout Inventory, General Health Questionnaire-12*
18	Milutinović D, Golubović B, Brkić N, Prokeš B	*Professional stress and health among critical care nurses in Serbia*	Croácia, 2012	Estudo transversal, nível V	*Nursing Stress Scale*
19	Zaghloul MS, Saquib J, AlMazrou A, Saquib N	*Mental Health Status of Expatriate Nurses in Northcentral Saudi Arabia*	Estados Unidos da América, 2019	Estudo transversal, nível V	*Depression, Anxiety and Stress Scale-21*
20	Kellogg MB, Knight M, Dowling JS, Crawford SL	*Secondary Traumatic Stress in Pediatric Nurses*	Estados Unidos da América, 2018	Estudo transversal, nível V	*Secondary Traumatic Stress Scale, Depression, Anxiety and Stress Scale-21*
21	Portero de la Cruz S, Vaquero Abellán M	*Professional burnout, stress and job satisfaction of nursing staff at a university hospital*	Brasil, 2015	Estudo transversal, nível V	*Nursing Stress Scale, Maslach Burnout Inventory, Cuestionario Font* Roja *de Satisfacción Laboral*
22	Mushtaq M, Sultana S, Imtiaz I	*The Trauma of Sexual Harassment and its Mental Health Consequences Among Nurses*	Paquistão, 2015	Estudo transversal, nível V	*Depression, Anxiety and Stress Scale-21*
23	Yoshizawa K, Sugawara N, Yasui-Furukori N, Danjo K, Furukori H, Sato Y, Tomita T, Fujii A, Nakagam T, Sasaki M, Nakamura K	*Relationship between occupational stress and depression among psychiatric nurses in Japan*	Estados Unidos da América, 2016	Estudo transversal, nível V	*General Health Questionnaire-12, Center for Epidemiologic Studies - Depression*
24	Karsavuran S, Kaya S	*The relationship between burnout and mobbing among hospital managers*	Inglaterra, 2017	Estudo transversal, nível V	*Leymann Inventory of Psychological Terrorization, Maslach Burnout Inventory*
25	Vandenbroeck S, Van Gerven E, De Witte H, Vanhaecht K, Godderis L	*Burnout in Belgian physicians and nurses*	Inglaterra, 2017	Estudo transversal, nível V	*Nursing Work Index-Revised, The Ways of Coping Checklist, Maslach Burnout Inventory*
26	Ceravolo D, Raines DA	*The Impact of a Mindfulness Intervention for Nurse Managers*	Estados Unidos da América, 2019	Estudo transversal, nível V	*Professional Quality of Life Scale*
27	Silva AA, Souza JM, Fischer FM	*Health-related quality of life and working conditions among nursing providers*	Brasil, 2010	Estudo transversal, nível V	*Job Satisfaction Scale, Effort-Reward Imbalance, Short-Form Health Survey-36*
28	Qin Z, Zhong X, Ma J, Lin H	*Stressors affecting nurses in China*	Estados Unidos da América, 2016	Estudo transversal, nível V	*Nursing Stress Scale*
29	Kawaguchi Y, Toyomasu K, Yoshida N, Baba K, Uemoto M, Minota S	*Measuring job stress among hospital nurses: an attempt to identify biological markers*	Japão, 2007	Estudo transversal, nível V	*Job Satisfaction Scale, Nursing Stress Scale*
30	Ramos FRS, Brehmer LCF, Dalmolin GL, Silveira LR, Schneider DG, Vargas MAO	*Association between moral distress and supporting elements of moral deliberation in nurses*	Brasil, 2020	Estudo transversal, nível V	*Moral Distress Scale*
31	Jang MH, Gu SY, Jeong YM	*Role of Coping Styles in the Relationship Between Nurses’ Work Stress and Well-Being Across Career*	Estados Unidos da América, 2019	Estudo transversal, nível V	*Nursing Stress Scale, The Ways of Coping Checklist Scale, Ryff’s Psychological Wellbeing Scale*
32	Liu S, Zhao N, Xie Y, Wang S, Ouyang X, Guo F, Gong Z, Zhou Q, Li J	*Psychological status of the staff in a general hospital during the outbreak of coronavirus disease 2019 and its influential factors*	China, 2020	Estudo transversal, nível V	*Depression, Anxiety and Stress Scale-21, The Ways of Coping Checklist Scale, Professional Quality of Life Scale*
33	Asgari S, Shafipour V, Taraghi Z, Yazdani-Charati J	*Relationship between moral distress and ethical climate with job satisfaction in nurses*	Inglaterra, 2019	Estudo transversal, nível V	*Moral Distress Scale, Job Satisfaction Scale*
34	Fagin L, Carson J, Leary J, De Villiers N, Bartlett H, O’Malley P, West M, McElfatrick S, Brown D	*Stress, coping and burnout in mental health nurses: findings from three research studies*	Inglaterra, 1996	Estudo transversal, nível V	*General Health Questionnaire-12, Maslach Burnout Inventory, De Villiers, Carson & Leary Stress Scale, The Ways of Coping Checklist Scale*
35	Pinikahana J, Happell B	*Stress, burnout and job satisfaction in rural psychiatric nurses: a Victorian study*	Austrália, 2004	Estudo transversal, nível V	*Maslach Burnout Inventory, Nursing Stress Scale, Job Satisfaction Scale*
36	Wang J, Okoli CTC, He H, Feng F, Li J, Zhuang L, Lin M	*Factors associated with compassion satisfaction, burnout, and secondary traumatic stress among Chinese nurses in tertiary hospitals: A cross-sectional study*	Inglaterra, 2020	Estudo transversal, nível V	*Professional Quality of Life Scale*
37	Ohnishi K, Kitaoka K, Nakahara J, Välimäki M, Kontio R, Anttila M	*Impact of moral sensitivity on moral distress among psychiatric nurses*	Inglaterra, 2019	Estudo transversal, nível V	*Moral Sensitivity Questionnaire, Moral Distress Scale*
38	Chang EM, Bidewell JW, Huntington AD, Daly J, Johnson A, Wilson H, Lambert VA, Lambert CE	*A survey of role stress, coping and health in Australian and New Zealand hospital nurses*	Inglaterra, 2007	Estudo transversal, nível V	*Nursing Stress Scale, The Ways of Coping Checklist Scale, Short-Form Health Survey-36*
39	Sawatzky JA	*Stress in critical care nurses: actual and perceived*	Estados Unidos da América, 1996	Estudo transversal, nível V	*Critical Care Nursing Stress Scale, Perceived Stress Scale*
40	Fukuda H, Ichinose T, Kusama T, Yoshidome A, Anndow K, Akiyoshi N, Shibamoto T	*The relationship between job stress and urinary cytokines in healthy nurses: a cross-sectional study*	Estados Unidos da América, 2008	Estudo transversal, nível V	*Nursing Stress Scale, Profile of Mood States*

Foram utilizados nos estudos 25 instrumentos de avaliação de condições emocionais (Quadro 3), sendo a maioria desses instrumentos elaborados e validados da década de 90 (n=12). Os instrumentos mais encontrados nos estudos com profissionais de enfermagem, atuantes em ambiente hospitalar, foram: o *Nursing Stress Scale* (NSS) (n=9); o *Maslach Burnout Inventory* (MBI) (n=6); o *Job Satisfaction Scale* (JSS) e o *The Ways of Coping Checklist* (WCCL) (ambos com n=5); o *Depression, Anxiety and Stress Scale-21* (DASS-21) e o *Professional Quality of Life Scale* (*ProQol*) (ambos com n=4); o *Moral Distress Scale* (MDS) e o *General Health Questionnaire-12* (GHQ-12) (ambos com n=3); e o *Short-Form Health Survey-36* (SF-36) (n=2). Uma parcela dos estudos^(15,17,19,20,23,27,29,31,37)^ aplicou um único instrumento para o alcance dos seus objetivos (n=9). Os instrumentos utilizados isoladamente foram o ProQol, MDS, NSS e DASS-21. Em relação à estrutura dos instrumentos, a maioria apresenta natureza multidimensional (n=20). Todos os instrumentos utilizam a escala tipo Likert como método de resposta.

**Quadro 3:** Instrumentos utilizados nos estudos desta pesquisa, Montes Claros, Minas Gerais, Brasil, 2025

	Instrumento	Quantidade utilização	Ano de elaboração	País de origem	Quantidade das dimensões estruturais	Quantidade de itens a serem respondidos	Áreas de avaliação
1	*Nursing Stress Scale*	9	1974	Canadá	2	34	Estresse: carga trabalho, morte e morrer, preparação, apoio colegas, tratamento incerto, conflito médicos, conflito enfermagem. Organização do trabalho.
2	*Maslach Burnout Inventory*	6	1981	Estados Unidos da América	3	22	Exaustão emocional, despersonalização, realização pessoal no trabalho.
3	*Job Satisfaction Scale*	5	1985	Estados Unidos da América	9	36	Remuneração, promoção, supervisão, benefícios, recompensas, condições operacionais, colaboradores, natureza do trabalho e comunicação.
4	*The Ways of Coping Checklist*	5	1980	Estados Unidos da América	2	66	Enfrentamento focalizado no problema (*problem-focused coping*): planejamento: desenvolver um plano de ação para lidar diretamente com o problema. Resolução ativa de problemas: tomar medidas concretas para resolver ou mitigar a situação estressante. Busca de suporte instrumental: procurar ajuda prática ou orientação para lidar com o problema/enfrentamento focado na emoção (*emotion-focused coping*). Busca de apoio emocional: procurar empatia, compreensão ou apoio emocional de outras pessoas. Aceitação: aceitar a realidade da situação estressante, sem necessariamente tentar mudá-la. Supressão de atividade: reduzir atividades ou distrações para lidar com a carga emocional.
5	*Depression, Anxiety and Stress Scale-21*	4	1995	Austrália	3	21	Depressão (sentimentos de tristeza, desesperança, falta de interesse ou prazer nas atividades cotidianas, entre outros sintomas associados à depressão), ansiedade (experiências de nervosismo, inquietação, preocupação excessiva, medo ou apreensão em relação ao futuro) e estresse (capacidade de relaxamento, irritabilidade, impaciência, sentimentos de sobrecarga ou sobrecarregamento).
6	*Professional Quality of Life Scale*	4	1995	Estados Unidos da América	3	28	Satisfação por compaixão (itens relacionados à satisfação no trabalho, sentido de propósito e realização pessoal), fadiga por compaixão (exaustão emocional e psicológica resultante do envolvimento contínuo em situações de sofrimento e trauma. Inclui itens como sintomas de estresse, esgotamento e desgaste emocional) e *burnout.*
7	*Moral Distress Scale*	3	1984	Estados Unidos da América	3	38	Causas, manifestações, consequências morais. Conflito ético, impotência e frustração, desconforto moral, dilemas éticos recorrentes, autonomia profissional.
8	*General Health Questionnaire-12*	3	1979	Reino Unido	3	12	Disfunção social, ansiedade e depressão, perda de confiança.
9	*Short-Form Health Survey-36*	2	1992	Estados Unidos da América	8	36	Capacidade funcional, limitações por aspectos físicos, dor, estado geral de saúde, vitalidade, aspectos sociais, limitações por aspectos emocionais, saúde mental.
10	*Critical Care Nursing Stress Scale*	1	1996	Canadá	4	40	Relações interpessoais, cuidado ao paciente, conhecimentos e habilidades, ambiente físico de trabalho.
11	*Moral Sensitivity Questionnaire*	1	1993	Suécia	6	30	Orientação interpessoal, significado da estrutura moral, expressando benevolência, autonomia modificada, experimentando conflito moral, confiança no conhecimento médico.
12	*Job Content Questionnaire*	1	1979	Estados Unidos da América	3	49	Demandas psicológicas, controle de decisões, apoio social no trabalho.
13	*Nursing Work Index-Revised*	1	2001	Estados Unidos da América	6	60	Autonomia, controle sobre o ambiente de trabalho, apoio da administração, relações de trabalho, satisfação com o trabalho, intenção de deixar o emprego.
14	*Leymann Inventory of Psychological Terrorization*	1	1990	Suécia	1	45	O instrumento avalia a exposição a comportamentos de terrorização psicológica no local de trabalho. As áreas de avaliação incluem diversos comportamentos de terrorização psicológica, tais como isolamento social, difamação, discriminação e outros tipos de assédio psicológico.
15	*Secondary Traumatic Stress Scale*	1	1995	Estados Unidos da América	3	17	Sintomas intrusivos, sintomas evitativos, sintomas de ativação.
16	*Impact Event Scale*	1	1997	Estados Unidos da América	3	22	Intrusão (refere-se à presença de pensamentos indesejados e imagens relacionados ao evento traumático), evitação (avalia comportamentos de evitação, como evitar pensamentos e sentimentos relacionados ao evento), ativação (mede sintomas de hiperativação, como irritabilidade, dificuldade de concentração e hipervigilância).
17	*De Villiers, Carson & Leary Stress Scale*	1	1996	Estados Unidos da América	3	30	Exaustão emocional, despersonalização, realização pessoal.
18	*Effort-Reward Imbalance*	1	1996	Alemanha	3	16	Esforço (*effort*): avalia o nível de demanda psicológica e física percebida no trabalho. Recompensas (*rewards*): mede as recompensas percebidas, incluindo salários, promoções, reconhecimento e segurança no emprego. Desequilíbrio (*imbalance*): reflete a comparação entre o esforço investido e as recompensas recebidas.
19	*Self-Rating Anxiety Scale*	1	1971	Estados Unidos da América	1	20	Gravidade dos sintomas de ansiedade.
20	*Center for Epidemiologic Studies - Depression*	1	1977	Estados Unidos da América	1	20	Avalia uma variedade de sintomas relacionados à depressão, incluindo sentimentos de tristeza, falta de interesse, perda de apetite e dificuldades de sono.
21	*Fear of COVID-19 Scale*	1	2020	Turquia	1	7	Preocupações com a saúde: avalia o medo relacionado à própria saúde e à possibilidade de contrair a COVID-19. Ansiedade sobre o contágio: explora a preocupação e a ansiedade em relação à possibilidade de transmitir ou contrair o vírus, seja para si mesmo ou para os outros. Impacto psicológico geral: inclui itens que abordam o impacto emocional mais amplo da pandemia, como ansiedade, preocupação constante e estresse psicológico. Medo de morrer devido à COVID-19: foca especificamente no medo de morte associado à infecção pelo vírus.
22	*Perceived* *Stress Scale*	1	1983	Estados Unidos da América	1	14	Sensação de imprevisibilidade: avalia o grau em que a vida do indivíduo é percebida como imprevisível ou incontrolável. Sentimento de controle: aborda a percepção do indivíduo em relação ao controle que ele acredita ter sobre as situações da vida. Sobrecarga percebida: refere-se à percepção de sobrecarga de demandas, responsabilidades ou pressões na vida cotidiana.
23	*Profile of Mood States*	1	1971	Estados Unidos da América	6	65	Tensão-ansiedade: avalia o grau de nervosismo, tensão e ansiedade que a pessoa está experimentando. Depressão-desânimo: mede sentimentos de tristeza, desânimo e desesperança. Irritabilidade-raiva: avalia a propensão da pessoa para ficar irritada, zangada ou frustrada. Vigor-atividade: refere-se à energia e disposição física e mental. Fadiga-cansaço: avalia o nível de fadiga e cansaço percebido. Confusão-desorientação: mede o grau de confusão, desorientação e falta de clareza mental.
24	*Cuestionario* *Font Roja de Satisfacción Laboral*	1	1988	Espanha	9	24	Satisfação com o trabalho, tensão no trabalho, competência profissional, pressão no trabalho, promoção profissional, relação interpessoal com os superiores, relação interpessoal com os colegas de trabalho, características extrínsecas de status o social e monotonia no trabalho.
25	*Ryff’s Psychological Wellbeing Scale*	1	1980	Estados Unidos da América	6	84	Autonomia: avalia o senso de independência e autodeterminação. Relações positivas com os outros: envolvimento positivo com os outros e relações interpessoais saudáveis. Domínio ambiental: senso de mestria e competência em gerenciar o ambiente. Crescimento pessoal: busca contínua por crescimento e desenvolvimento pessoal. Propósito na vida: ter objetivos significativos e um senso claro de direção na vida. Autoaceitação: aceitação e respeito por si mesmo, incluindo aspectos positivos e negativos.

Na análise do conteúdo das dimensões e itens de avaliação dos instrumentos, emergiram cinco agrupamentos de semelhança codificada como: bem-estar do profissional; estresse e *burnout*; satisfação e insatisfação no trabalho; estratégias de enfrentamento; condições de trabalho e relações interpessoais.

## DISCUSSÃO

Os estudos revisados apontam para vários fatores importantes para o entendimento da situação emocional de profissionais de enfermagem. Algumas discussões desses achados serão sistematizadas para o entendimento dessa temática, que é muito fundamentada na utilização de instrumentos para sua mensuração, uma vez que utilizar instrumentos validados para entendimento de circunstâncias subjetivas, como as emocionais, torna-se imprescindível para seu entendimento e análise.

Os EUA ganham destaque em local de publicação de artigos da área. Essa característica vai de encontro a fatores relacionados à sua cultura científica, social e econômica. Historicamente, os EUA lideram o fomento em pesquisas na área da saúde por meio de incentivos financeiros e grande número de meios de divulgação científica^(43)^. Esse fomento pode estar relacionado a vertentes que vão desde a construção contemporânea do nacionalismo americano, fundamentado em políticas como *America First* ^(44)^, até preocupações econômicas com o custo do sistema de saúde americano. A enfermagem é a maior categoria de um sistema de saúde. Assim, constante monitoramento emocional significa menos afastamentos e prejuízos na prestação de serviço em saúde^(45)^.

Os resultados da revisão apontam que os dez anos anteriores a esta pesquisa (2014-2023) tiveram muita representativa na aplicação de instrumentos de avaliação emocional em profissionais de enfermagem em ambiente hospitalar. Vários são os eventos que contribuem para o aumento de pesquisas na área da saúde mental em profissionais de enfermagem. Devemos iniciar citando o avanço, neste período, da complexidade assistencial a que esses profissionais estão submetidos na produção do seu serviço, o que aumenta a sobrecarga de trabalho, sentimentos ansiosos e estresse^(46)^. Além disso, todas essas situações foram potencializadas com o surgimento da pandemia de COVID-19 ao final de 2019, que acarretou sérios eventos emocionais em profissionais de enfermagem de todo o mundo^(47)^. Tal evento é tão importante para essa temática que culminou na elaboração de instrumento próprio para avaliar as condições emocionais de profissionais de saúde tratando de pacientes com esse diagnóstico: o *Fear of COVID-19 Scale* ^(14)^.

Os estudos utilizaram, em sua maioria, instrumentos elaborados na década de 90. Essa década é marcada no Brasil e no mundo por evoluções teóricas, diagnósticas e ideológicas, o que contribui diretamente para o surgimento de preocupações com a saúde mental e consequente surgimento de instrumentos. A desmistificação e redução do estigma para pacientes em transtorno mental, os avanços em pesquisas e tratamento na área, o ativismo e a defesa dos pacientes (no Brasil, destaca-se a enorme discussão da Reforma Psiquiátrica), e o aumento das possibilidades de diagnóstico foram pilares fundamentais para essa ocorrência^(48)^. Todos esses movimentos impactaram não apenas a assistência em saúde mental, mas também as avaliações das condições emocionais em profissionais de saúde.

Esta revisão resultou em uma série de instrumentos utilizados para avaliar condições emocionais em profissionais de enfermagem no ambiente hospitalar. Em destaque, temos o NSS, específico para a área da enfermagem, o MBI (n=6), o JSS, o DASS-21 e o ProQol (ambos com n=4). Destacaram instrumentos específicos para a equipe de enfermagem. Instrumentos validados exclusivamente para categorias se tornaram fundamentais para entender o fenômeno em populações específicas^(49)^. Somente instrumentos especializados conseguem mensurar realidades únicas não evidenciadas em contextos generalistas.

A análise de conteúdo das dimensões e consequente natureza dos instrumentos foi essencial para o entendimento dos contextos e universos que são considerados para avaliar as condições emocionais dos profissionais de enfermagem. Emergiram agrupamentos de semelhança codificada, que detalharemos a partir deste momento para compreensão mais apurada.

Instrumentos mais voltados para o bem-estar do profissional são considerados multidimensionais, ou seja, são instrumentos que consideram que questões emocionais são consequências de diversos fatores, internos e externos à pessoa. Na contemporaneidade, as evidências para abordagens em questões emocionais ressaltam a importância de visões e ações de forma integrada e multidimensional^(50)^. Vários foram os estudos^(15,17,19,20,23,27,28,32,37,39)^ que utilizaram esse tipo de instrumento, com dimensões que abordam diferentes áreas no enfoque.

Os instrumentos revelados para essa categoria são o ProQol, o SF-36, o *Ryff’s Psychological Wellbeing Scale*, o NSS e o DASS-21. Acrescenta-se que o enfoque multidimensional dessa categoria de instrumento permitiu estudos^(15,17,19,20,23,27,29,37)^ que utilizaram um único instrumento. Estes estudos consideraram que sua aplicação isolada revela resultados suficientes para análise da natureza das questões emocionais.

Outro agrupamento foi o “estresse e *burnout*”. Nessa categoria temática, destacam-se os instrumentos que voltaram seus resultados para uma análise das consequências mais esperadas de situações emocionais depreciativas, situações de estresse e *burnout*. Profissionais que atuam em unidades hospitalares, ambiente com alta demanda psicológica e baixo controle sobre essas, estão constantemente em *burnout* e estresse ocupacional^(46)^.

Os instrumentos que atendem esse propósito são o MBI, o MDS, o GHQ-12 e o WCCL. Estes instrumentos foram utilizados em estudos^(18,22,25,26,31,34,35,36,38)^ que, entre outros objetivos, buscavam detectar evidências de questões emocionais instauradas (no caso, estresse e *burnout*). Situações emocionais depreciativas, como estresse e *burnout*, em profissionais de enfermagem, trazem impactos catastróficos em diversas dimensões, seja na vida pessoal do profissional, em sua manutenção no mercado de trabalho ou na segurança do paciente^(13)^.

A “satisfação e insatisfação no trabalho” foi outro agrupamento de instrumentos que emergiu de determinados estudos analisados^(14,16,22,26,28,36)^. Engloba instrumentos capazes de qualificar aspectos de satisfação dos profissionais de enfermagem hospitalar na execução de suas atividades, ou seja, instrumentos capazes de qualificar fenômenos presentes na execução do serviço de enfermagem ao paciente hospitalar que geram alterações emocionais. Os determinantes da satisfação ou da insatisfação no trabalho são diversos, multifatoriais e dinâmicos, sofrendo influência do ambiente, das condições de trabalho e das características que o trabalhador tem para enfrentar as adversidades do meio. A insatisfação se relaciona à vivência de inadaptação entre os componentes mental e físico, ao sofrimento, à ansiedade e às dificuldades na organização do trabalho, nem sempre explicitadas pelo trabalhador. Já a satisfação tem relação com o significado e o valor atribuído ao trabalho pelo indivíduo, com a capacidade de adaptação ao conteúdo das atividades produtivas, as relações interpessoais, a qualidade da tarefa prestada e o bem-estar^(50)^.

Os instrumentos que enquadram nesta categoria foram o JSS, o *Nursing Work Index-Revised* (NWI-R), o *Job Content Questionnaire* (JCQ) e o *Cuestionario Font Roja de Satisfacción Laboral*.

Outra categoria emergente da análise do conteúdo das dimensões dos instrumentos nos estudos^(26,32,39)^ foi a “estratégias de enfrentamento”. Essa categoria é importante, porque aponta as alterações emocionais decorrentes de mecanismos para enfrentar, controlar ou minimizar as consequências das situações de ansiedade e estresse já presentes nos trabalhadores de enfermagem do ambiente hospitalar. O instrumento que se enquadrou nessa categoria foi o WCCL. Atente-se que este instrumento também emergiu na categoria estresse e *burnout*, visto que é um instrumento que aponta efeitos emocionais e verifica métodos de enfrentamento. Como característica de enfrentamento verificada pelo instrumento, podemos destacar a resiliência e o *coping*.

A resiliência resulta da interação entre atributos individuais e ambientais (familiar, social, cultural) para uma capacidade de resposta e superação. A resiliência abarca modalidades de resistência ao estresse, ao passo que o *coping* representa a capacidade de enfrentá-lo e de promover uma adaptação positiva. O *coping* ocorre em um determinado momento, e a resiliência, ao longo do tempo. Profissionais de enfermagem com maiores níveis de autoeficácia e resiliência são menos propensos a desenvolver transtornos mentais, como depressão e *burnout* ^(39)^.

Outra categoria deste estudo para o entendimento dos instrumentos foi a “condições de trabalho e relações interpessoais”. Essa leva em consideração, entre outros fatores, situações externas do profissional no ambiente de trabalho, como a estrutura física, o apoio social e as relações interpessoais entre a equipe de enfermagem e outras categorias que compõem o ambiente de prestação de serviço hospitalar. Várias são as situações externas que depreciam as condições emocionais dos profissionais de enfermagem. A sobrecarga de trabalho, a falta efetiva de condições estruturais, tecnológicas e a dificuldade de comunicação são apontadas como pontos a serem investigados.

Ações devem focar nesses contextos, e uma comunicação efetiva entre trabalhadores pode fazer a diferença no ambiente de trabalho e na saúde mental, posto que potencializa o trabalho em equipe, a participação em decisões nos fluxos operacionais e o apoio entre os colegas^(47)^. Alguns estudos^(22,26,40,41)^ utilizaram os instrumentos desta categoria, como o *Critical Care* NSS, NWI-R e o JCQ, sendo que os dois últimos também estiveram presentes na categoria satisfação e insatisfação no trabalho.

Os achados desta revisão evidenciam que, embora exista diversidade de instrumentos para mensuração emocional em enfermeiros hospitalares, a maioria não foi desenvolvida especificamente para esta categoria profissional, representando a principal lacuna identificada neste estudo.

Os instrumentos mais frequentemente utilizados, como NSS, MBI, DASS-21 e ProQol, abrangem múltiplas dimensões emocionais e podem ser aplicados isoladamente ou em conjunto, dependendo dos objetivos da avaliação. Essa síntese contribui para orientar pesquisadores e gestores na escolha de ferramentas adequadas para avaliação da saúde mental de profissionais de enfermagem, ao mesmo tempo em que evidencia a necessidade urgente de desenvolvimento e validação de instrumentos específicos que capturem as particularidades do trabalho de enfermagem no contexto hospitalar.

A disponibilidade de instrumentos específicos e culturalmente adaptados é essencial para subsidiar políticas institucionais de prevenção e intervenção, promover ambientes de trabalho saudáveis e, consequentemente, melhorar a qualidade e a segurança da assistência prestada aos pacientes.

### Limitações do estudo

As limitações desta revisão incluem a restrição a artigos publicados nos idiomas português, inglês e espanhol, o que pode ter resultado na exclusão de estudos relevantes em outras línguas. Adicionalmente, a exclusão de literatura cinzenta, embora justificada pelo foco em instrumentos publicados em periódicos revisados por pares, pode ter limitado a identificação de ferramentas em fase de validação. Por fim, o processo de análise de conteúdo, ainda que conduzido com rigor, possui um componente interpretativo inerente, cujo viés foi minimizado pela análise independente por múltiplos revisores.

### Contribuições para a área da enfermagem, saúde ou políticas públicas

O presente estudo contribui de maneira significativa para a área da enfermagem, da saúde e das políticas públicas, ao sistematizar e evidenciar os instrumentos validados mais utilizados para avaliar as condições emocionais de profissionais de enfermagem no contexto hospitalar. Ao mapear categorias temáticas como estresse, *burnout*, satisfação no trabalho, estratégias de enfrentamento e condições laborais, a pesquisa permite a identificação de dimensões críticas que impactam diretamente o bem-estar dos profissionais e, por conseguinte, a qualidade da assistência prestada aos pacientes. Tais achados não apenas fortalecem a fundamentação teórica e metodológica da enfermagem enquanto ciência preocupada com a saúde mental de seus profissionais, mas também fornecem subsídios técnicos para a formulação de políticas institucionais mais eficazes. Políticas públicas de saúde voltadas à prevenção do adoecimento mental, ao fortalecimento da resiliência e à promoção de ambientes de trabalho saudáveis podem se apoiar nos instrumentos e evidências aqui discutidos, resultando em medidas estratégicas de gestão do trabalho e valorização da força de trabalho da enfermagem, a maior categoria profissional da saúde. Assim, este estudo reafirma a relevância da avaliação emocional sistemática como eixo norteador para decisões clínicas, administrativas e políticas.

## CONCLUSÕES

Esta revisão de escopo identificou 25 instrumentos de mensuração de estados emocionais aplicados em profissionais de enfermagem hospitalar, mapeados a partir de 28 estudos. Os instrumentos mais frequentemente utilizados foram o NSS, o MBI, o DASS-21 e o ProQOL. A análise revelou cinco categorias temáticas principais: bem-estar profissional; estresse e *burnout*; satisfação laboral; estratégias de enfrentamento; e condições de trabalho/relações interpessoais.

O principal achado é a predominância de instrumentos genéricos não desenvolvidos especificamente para as particularidades da prática de enfermagem, o que representa uma lacuna importante na literatura. A maioria das ferramentas é multidimensional e data da década de 1990, indicando uma necessidade de atualização e desenvolvimento de novos instrumentos que considerem os contextos de trabalho contemporâneos.

Os resultados reforçam a necessidade de futuras pesquisas focadas no desenvolvimento e validação de instrumentos específicos para a enfermagem que possam capturar de forma mais precisa os desafios emocionais da profissão. Ferramentas adequadas são essenciais para subsidiar a criação de intervenções eficazes, promover ambientes de trabalho saudáveis e, consequentemente, melhorar a qualidade do cuidado e a segurança do paciente, em alinhamento com as metas do ODS 3.

## Data Availability

Os dados de pesquisa estão disponíveis no corpo do artigo.
